# Cytosolic CTP Production Limits the Establishment of Photosynthesis in *Arabidopsis*

**DOI:** 10.3389/fpls.2021.789189

**Published:** 2021-11-30

**Authors:** Leo Bellin, Vanessa Scherer, Eva Dörfer, Anne Lau, Alexandre Magno Vicente, Jörg Meurer, Daniel Hickl, Torsten Möhlmann

**Affiliations:** ^1^Plant Physiology, Faculty of Biology, University of Kaiserslautern, Kaiserslautern, Germany; ^2^Plant Molecular Biology, Faculty of Biology, Ludwig-Maximilians-University Munich, Planegg-Martinsried, Germany

**Keywords:** pyrimidine *de novo* synthesis, CTP, photosynthesis, chloroplast, genome, deoxycytidine

## Abstract

CTP synthases (CTPS) comprise a protein family of the five members CTPS1-CTPS5 in Arabidopsis, all located in the cytosol. Specifically, downregulation of *CTPS2* by amiRNA technology results in plants with defects in chlorophyll accumulation and photosynthetic performance early in development. CTP and its deoxy form dCTP are present at low levels in developing seedlings. Thus, under conditions of fast proliferation, the synthesis of CTP (dCTP) can become a limiting factor for RNA and DNA synthesis. The higher sensitivity of *ami-CTPS2* lines toward the DNA-Gyrase inhibitor ciprofloxacin, together with reduced plastid DNA copy number and 16S and 23S chloroplast ribosomal RNA support this view. High expression and proposed beneficial biochemical features render CTPS2 the most important isoform for early seedling development. In addition, CTPS2 was identified as an essential enzyme in embryo development before, as knock-out mutants were embryo lethal. In line with this, *ami-CTPS2* lines also exhibited reduced seed numbers per plant.

## Introduction

Nucleotides are essential components of all living organisms. As building blocks of DNA and RNA, they are involved in the storage and transmission of genetic information. In addition, nucleotides function as energy transmitters for many other metabolic pathways. Further, they act as cofactors for the synthesis of sugars and polysaccharides, glycoproteins, and phospholipids (Moffatt and Ashihara, 2002; Zrenner et al., 2006).

The maintenance of the nucleotide pool is achieved by the interaction of *de novo* synthesis, salvage, and degradation. Plant pyrimidine *de novo* biosynthesis consists of six enzymatic steps distributed over the plastid, cytosol, and mitochondrion, ending in the cytosol with the formation of uridine-monophosphate (UMP; Zrenner et al., 2006; Witz et al., 2012). UMP can be further phosphorylated to form uridine- di- (UDP) and triphosphates (UTP). Thereby, it has been shown that aspartate transcarbamoylase (ATC) is one of the major regulated enzymes for *de novo* synthesis of UMP in plants. The enzyme is localized in the chloroplast and catalyzes the condensation of carbamoyl aspartate from aspartate and carbamoyl phosphate (Zrenner et al., 2006; Bellin et al., 2021).

Another limiting step of the synthesis of nucleotides involves the amination of UTP to form cytidine triphosphate (CTP) further used for the synthesis of RNA and as deoxynucleotide for DNA. This enzymatic step is catalyzed by the CTP synthase (CTPS) gene family, comprising of five members in Arabidopsis, all of these proteins localize in the cytosol (Moffatt and Ashihara, 2002; Zrenner et al., 2006; Witz et al., 2012; Daumann et al., 2018). CTPS belongs to a family of enzymes that are highly conserved throughout all kingdoms of life (Zhou et al., 2020). The activity of the enzyme is regulated in various ways. Besides post-translational modifications (phosphorylation), the enzymatic activity is regulated by allosteric binding of GTP and feedback inhibition by its product CTP (Levitzki and Koshland, 1972; Chang and Carman, 2008). Another special type of regulation is the formation of filaments, which was studied in several organisms, such as *Escherichia coli*, *Saccharomyces cerevisiae*, *Drosophila melanogaster*, and *Homo sapiens* (Liu, 2010; Barry et al., 2014; Noree et al., 2014; Lynch et al., 2017). Arabidopsis CTPS3 to 5 can form filaments as shown by transient expression of corresponding YFP fusion proteins, whereas CTPS1 and 2 always appeared soluble (Daumann et al., 2018; Alamdari et al., 2021).

All five isoforms show high similarity in amino acid sequence but exhibit different tissue-specific expression patterns in Arabidopsis (Zimmermann et al., 2004). Among the five isoforms, only the single knock-out *ctps2* showed an obvious phenotype and was unable to produce viable progeny (Daumann et al., 2018). Further studies demonstrated that CTPS2 is crucial for proper embryo and seed development, as well as early stages of plant growth (Hickl et al., 2021). The aim of this work was to investigate whether CTPS2 has additional roles in vegetative development in Arabidopsis.

## Materials and Methods

### Plant Growth

For DNA isolation, tissue collection, and phenotypic inspection, WT and transgenic *Arabidopsis thaliana* (L.) Heynh. plants (ecotype Columbia) were used throughout. Plants were grown on standardized ED73 soil (Einheitserde und Humuswerke Patzer) or on agar plates either under short day (SD) conditions with 10h light and 14h darkness. For growth, we used light intensity of 120μmol quanta m^−2^s^−1^, 22°C, and 60% humidity. Illumination was done with LED light (Valoya NS1, Valoya, Finnland).

For growth experiments on sterile agar plates, seeds were surface sterilized before placing the seeds on half-strength MS, supplemented with 0.1% (w/v) sucrose. Prior to germination, seeds were incubated for 24h in the dark at 4°C for imbibition (Weigel and Glazebrook, 2002). If not stated otherwise, plant material was harvested in the middle of the light period and frozen in liquid nitrogen for further use.

### Construction of CTPS2 Knock-Down Plants

To generate CTPS2 (At3g12670) knock-down mutants, an established protocol for gene silencing by artificial microRNA (amiRNA) was used (Schwab et al., 2006). For the design of amiRNA targeting *CTPS2*, an online tool was used.^1^ Therefore, different primers with Gateway™ compatible sequences attP1 and attP2 were designed (Supplementary Table 2). All cloned fragments were subcloned *via* BP-clonase reaction into the Gateway™ entry vector pDONR/Zeo and later *via* LR-clonase reaction into the destination vector pK2GW7, which contains a 35S-CaMV promoter.

All constructs which were used for Arabidopsis transformation by floral dip (Narusaka et al., 2010) were previously transformed into *A. tumefaciens* strain GV3101 (Furini et al., 1994). Knock-down lines were selected by screening for different independent lines exhibiting 37–18% *CTPS2* transcript levels. Three of these lines (*ami-ctps2-1*, *ami-ctps2-2*, and *ami-ctps2-3*) were selected for further analysis.

Single knock-out lines for CTPS1,3,4, and 5 (*ctps1-1*, SALK_031868; *ctps3–1*, SALK_118507; *ctps4–1*, SALK_020074C; and *ctps5–1*, SAIL_645_D02) from the Salk and SAIL collection were described previously (Daumann et al., 2018). To analyze CPTS2-promotor expression, CTPS2::GUS lines were used which were created and described recently (Hickl et al., 2021).

### Chlorophyll Analysis

Chlorophyll was extracted from ground leave tissue with 80% ethanol. After boiling at 95°C for 10min and subsequent sedimentation of insoluble contents (10min 20,000 ´ *g*), chlorophyll was measured by the absorbance of the supernatant at 652nm. The calculation was performed as described by Arnon (1949). For each biological replicate, 50–100 7-day-old seedlings or whole rosettes from five 28-day-old plants were pooled.

### RNA Gel-Blot Hybridization and Gene Expression Analyses

RNA gel-blot analyses, total RNA isolation, electrophoresis, blotting, and hybridization with radioactive labeled probes were carried out as described recently (Manavski et al., 2015). Oligonucleotides and primers for PCR-probes are listed in Supplementary Table 2. RNA was extracted from leaf material of soil-grown plants, which was homogenized in liquid nitrogen prior to extraction of RNA with the Nucleospin RNA Plant Kit (Macherey-Nagel, Düren, Germany) according to the manufacturer’s advice. RNA purity and concentration were quantified using a NanoDrop spectrophotometer (Thermo Fischer Scientific). Total RNA was transcribed into cDNA using the qScript cDNA Synthesis Kit (Quantabio, United States). QPCR was performed using the Quantabio SYBR green quantification kit (Quantabio) on PFX96 system (BioRad, Hercules, CA, USA) using specific primers (Supplementary Table 2). Actin (At3g18780) was used as reference gene for transcript normalization.

### Measuring Organelle Genome Copy Number

Total genomic DNA was extracted from seven and 21-day-old plants. In brief, samples were grinded in liquid nitrogen before adding 500μl shorty buffer (0.2M TRIS/HCl pH 9.0; 0.4M LiCl; 25mM EDTA pH 8; and 1% SDS). After mixing, tubes were centrifuged for 10min at 20,000*g* to remove cellular debris. The supernatant (350μl) was transferred into a new tube containing 350μl isopropanol. After centrifugation (10min at 20.000*g*), the supernatant was removed, and the pellet was washed with 70% ethanol. The dried pellet was resolved in 25μl H_2_O and the isolated gDNA was stored until use at 4°C.

In order to quantify the organellar DNA amount, real-time qPCR was performed, using actin as a reference probe. Each qPCR reaction was performed using 10ng of genomic DNA. For nuclear and plastidic genome, three pairs of primers were used targeting three different genes (nuclear, *petC*, *Lhcb1.4* and *Lhcb2.3* and plastid, *rubiscoL*, *psaA*, and *psbA*). Organellar DNA copy numbers for the plastid were calculated as average levels of plastid/nuclear probes.

### Germination Assays and Root Growth Tests

Seed germination was analyzed with 38–42 seeds per genotype on a Petri dish. Each experiment was repeated three times. Seeds were grown on agar plates starting at the onset of light. After indicated time points, seeds were inspected for radicle protrusion.

For root growth, seeds were treated as indicated above and grown vertically on square (120×120mm) petri plates. A total of 35 seeds per genotype were inspected in parallel and the experiment was repeated twice. The root length of 7-day-old seedlings was measured after scanning of agar plates with help of ImageJ software.

### Supplementation and Genotoxic Treatments

To investigate whether cytidine or deoxycytidine influences plant growth, seeds were grown on 1/2 MS medium containing either 1mM cytidine or 1mM deoxycytidine. After 7days, root growth was measured with the help of ImageJ.

To first analyze the sensitivity of the CTPS knock-down lines compared to the WT to the genotoxic reagent CIP (CIP; Sigma-Aldrich, St. Louis, MO, United States), seeds were placed on 1/2 MS medium containing 0.50 and 0.25μM CIP, respectively. To analyze the influence of CIP on the expression of CTPS2 in WT genotypes, CTPS2::GUS promoter seeds were grown in presence of the same CIP concentrations indicated above and examined after 7days using GUS staining.

### GUS Staining for Promotor Activity

Tissue from transgenic plants was collected in glass vials, filled with ice-cold 90% acetone, and incubated for 20min at room temperature. Subsequently, the samples were stained according to standard protocols (Weigel and Glazebrook, 2002).

### Pulse-Amplitude-Modulation (PAM) Fluorometry Measurements

A MINI-IMAGING-PAM fluorometer (Walz Instruments, Effeltrich, Germany) was used for *in vivo* chlorophyll fluorescence measurements (Schreiber et al., 2007). Seven- and 21-day-old plants were dark adapted for 10min before the measurement. After two saturating light pulses in the dark, actinic light stepwise increased (PAR=1, 21, 41, 76, 134, 205, 249, 298, 371, 456, 581, and 726) every 20s without additional dark phases. Results were calculated and visualized with imaging win software (v2.41a; Walz Instruments, Effeltrich, Germany).

### Evans-Blue Staining

Seedlings were incubated with freshly prepared staining solution [0.25g Evans Blue (Sigma) in 100ml 0.1M CaCl_2_ (pH 5.6)] for 10min under vacuum in the dark. After washing three times with water or CaCl_2_, images of the stained seedlings were taken with a Leica MZ10 F microscope equipped with a Leica DFC420 C camera.

### Silique Analysis

Siliques of soil-grown plants were harvested 8–10days after fertilization (DAF). To decolorize the pods, they were incubated in 100% ethanol at 90°C for 10min. Images were taken with a Leica MZ10 F microscope equipped with a Leica DFC420 C camera.

### Accession Numbers

CTP Synthase 1 (atCTPS1; At1g30820); CTP Synthase 2 (AtCTPS2; At3g12670); CTP Synthase 3 (atCTPS3; at4g02120); CTP Synthase 4 (atCTPS4; At4g20320); CTP Synthase 5 (atCTPS5; At2g34890); Actin2 (Act2; At3g18780); petC (atPetC; At4g03280); Lhcb1.4 (atLHCb1.4; At2g34430); Lhcb2.3 (atLHCb2.3; At3g27690); psaA (atPsaA; AtCg00350); psaD (atPsaD1; At4g02770); and psbA (atPsbA; AtCg00020).

## Results

**Figure 1:**
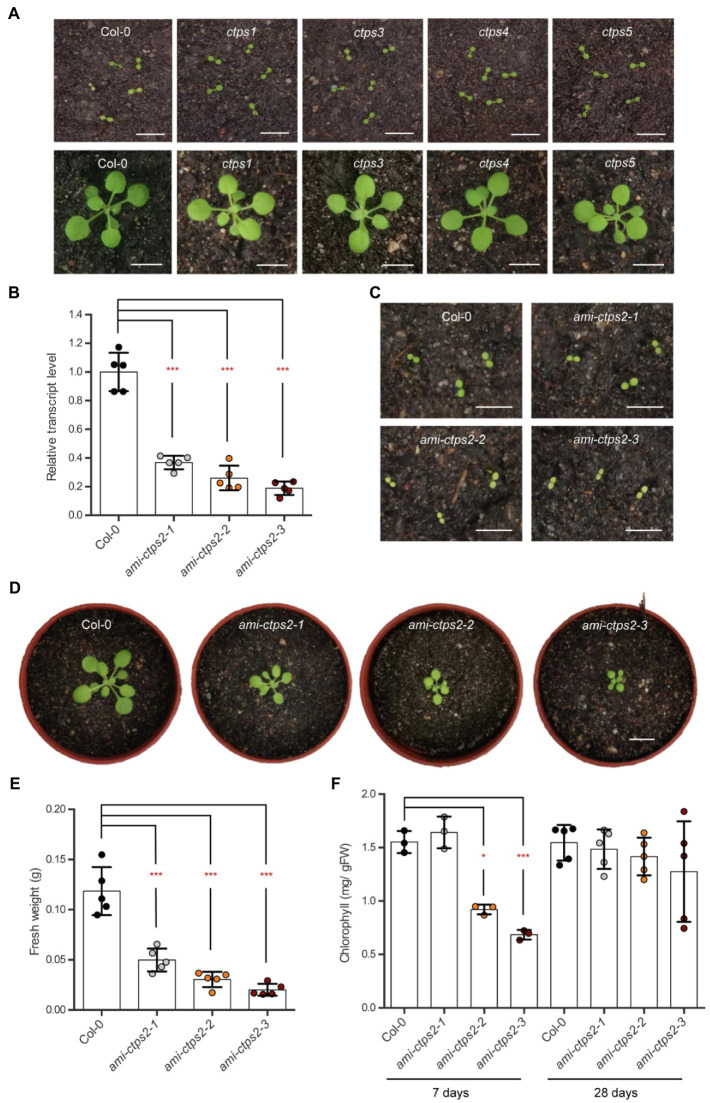
Silencing *CTPS2* inhibits plant growth. **(A)** Shown are 7-day-old *ctps*1, 3, 4, and 5 knock-out plants (top) and 21-day-old (bottom) *ctps*1, 3, 4, and 5 knock-out plants with the corresponding (Col-0) control plants. **(B)** Relative *CTPS2* transcript levels in *ami-CTPS2* lines and Col-0. Actin was used as a reference gene and WT was set to 1 (*n*=5). **(C)** Typical examples of 7-day-old CTPS2 knock-down plants and **(D)** 21-day-old CTPS2 knock-down plants raised under a 10h light/14h dark regime. **(E)** Fresh weight of 28-day-old plants (*n*=5) and **(F)** chlorophyll contents of 7days (*n*=3) and 28days (*n*=5) of growth. Plotted are the means of biological replicates +/− SD. For statistical analysis, one-way ANOVA was performed followed by Dunnett’s multiple comparison test (*p<0.05; ***p<0.001). Scale bar in A, C, and D=1cm.

Previous studies have shown that CTPS2 is required to complete embryo development. Even though Arabidopsis possesses five different isoforms, only *CTPS2* T-DNA insertion lines were unable to produce homozygous offspring (Daumann et al., 2018). The single knock-out mutant lines *ctps1*, *3*, *4*, and *5* did not show apparent phenotypical alterations after one (top; Figure 1A) and 3weeks of growth (bottom; Figure 1A). After 4weeks of growth, the fresh weight and rosette diameter were comparable to the wild type (WT; Supplementary Figures 1A–C).

Segregation analyses showed that heterozygous *+/ctps2* lines exhibited a seed abortion rate of about 25% which strongly indicates that CTPS2 loss of function is embryo lethal. These observations are supported by additional studies of the CTPS2 promoter, which showed a specific expression pattern during embryo development (Hickl et al., 2021).

Thus, to gain deeper insights into the role of CTPS2 in plant development, three independent knock-down mutant lines were generated. Using the artificial micro-RNA method, the transcript levels of CTPS2 were reduced to 36.8, 26, and 18.8% in the mutant lines *ami-ctps2-1*, *ami-ctps2-2*, and *ami-ctps2-3* compared to the control line (Figure 1B). In the course of this work, these knock-down lines, with different degrees of growth restrictions, were studied in more detail.

### A Reduced Transcript Level of CTPS2 Impairs Plant Growth and Development

Initial phenotypic studies of the mutant lines revealed that all three knock-down lines exhibited greatly reduced growth (Figures 1C,D; Supplementary Figures 1D,E).

A comparison of the mutant lines *ami-ctps2-1*, *ami-ctps2-2*, and *ami-ctps2-3* with the WT showed a reduction in fresh weight of 58, 74.5, and 83%, respectively (Figure 1E). This strong reduction was accompanied by a decreased rosette diameter (Supplementary Figures 1D,E). Closer inspection revealed that in the early stages of plant development *CTPS2* knock-down lines *ami-ctps2-2* and *ami-ctps2-3* showed a pale green phenotype (Figure 1C) due to reduced amounts of chlorophyll (Figure 1F), whereas after 4weeks of growth, no significant alterations in chlorophyll contents were observed (Figures 1D,F).

To examine whether reduction of CTPS2 expression affects transcript levels of the other CTPS isoforms, corresponding transcripts were quantified by quantitative real-time PCR. The expression of CTPS1 was increased in the *ami-ctps2-2* and *ami-ctps2-3* mutants but unaltered in *ami-ctps2-1*. This can be interpreted as an attempt to compensate CTPS2 loss when transcript levels drop below 30%.

No changes in transcript levels were detected for CTPS3, whereas CTPS4 was reduced in all three CTPS knock-down lines. The transcript content of CTPS5 was below the detection limit and could therefore not be examined (Supplementary Figure 1F). This observation can be explained by pollen specific expression of CTPS5 (Zimmermann et al., 2004).

### Seed Germination and Root Development Are Altered in CTPS2 Knock-Down Lines

**Figure 2:**
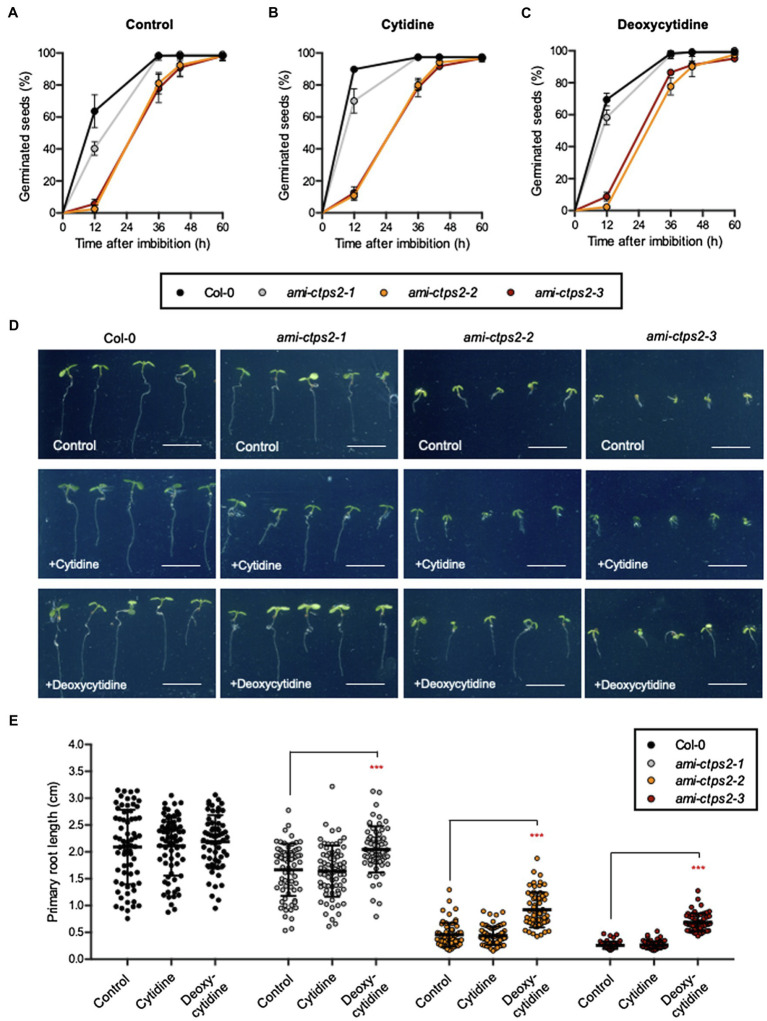
*CTPS2* knock-down mutants show delayed germination and development. Seed germination **(A–C)** was monitored in a time course of 60h after imbibition. Plants were grown under **(A)** control conditions on ½ MS media and supplemented with **(B)** 1mM cytidine or **(C)** 1mM deoxycytidine under a 10h light/14h dark regime. **(D,E)** Root growth was quantified on 6-day-old seedlings. Data points represent means ± SD. Asterisks depict significant changes between the different lines referring to the WT according to one-way ANOVA followed by Dunnett’s multiple comparison test (***=*p*<0.001). Scale bar in D=1cm.

In previous studies, it has been shown that CTPS2 is essential for embryo development (Daumann et al., 2018; Hickl et al., 2021). In order to investigate whether the phenotypes observed in Figure 1 are a result of delayed germination, the germination rate was examined over a time course of 60h after imbibition. The knock-down lines *ami-ctps2-2* and *ami-ctps2-3* showed delayed germination, whereas *ami-ctps2-1* developed almost like WT. After 36h, 100% of both WT and *ami-ctps2-1* seeds were germinated, whereas approximately 20% of *ami-ctps2-2* and *ami-ctps2-3* seeds had not yet germinated (Figure 2A).

In addition to the energy-consuming pyrimidine *de novo* synthesis and independent of CTPS activity, plants can recycle nucleosides *via* the salvage pathway. Therefore, we tested if either cytidine or deoxycytidine can restore normal germination and growth in the CTPS2 mutant lines. Here, neither cytidine (Figure 2B) nor deoxycytidine (Figure 2C) could abolish a delay in the germination of *ami-ctps2-2* and *ami-ctps2-3*.

After 1week of growth, all CTPS2 mutants showed a significant reduction in root length compared to the control plants. Under standard conditions, *ami-ctps2-2* and *ami-ctps2-3* grew very slowly. While the addition of cytidine showed no effect on root growth, supplementation of deoxycytidine resulted in enhanced root growth in all three mutant lines (Figures 2D,E).

### CTPS2 Is Required for Plastid Genome Integrity and Ribosomal RNA Synthesis

The pale green phenotype of the 7-day-old seedlings (Figure 1C) cannot be fully explained by delayed germination and thus, could be a hint toward disturbed chloroplast biogenesis, since chloroplasts depend on the import of nucleotides to maintain their genome (Witte and Herde, 2020).

Given that pyrimidine *de novo* synthesis ends in the cytosol, it has not yet been fully investigated how the chloroplast is supplied with nucleotides. One experimental approach to examine the role of CTPS2 in the maintenance of the plastid genome was a ciprofloxacin (CIP) treatment. CIP is a DNA-Gyrase inhibitor that induces the formation of double-strand breaks in organellar DNA (Evans-Roberts et al., 2016).

**Figure 3:**
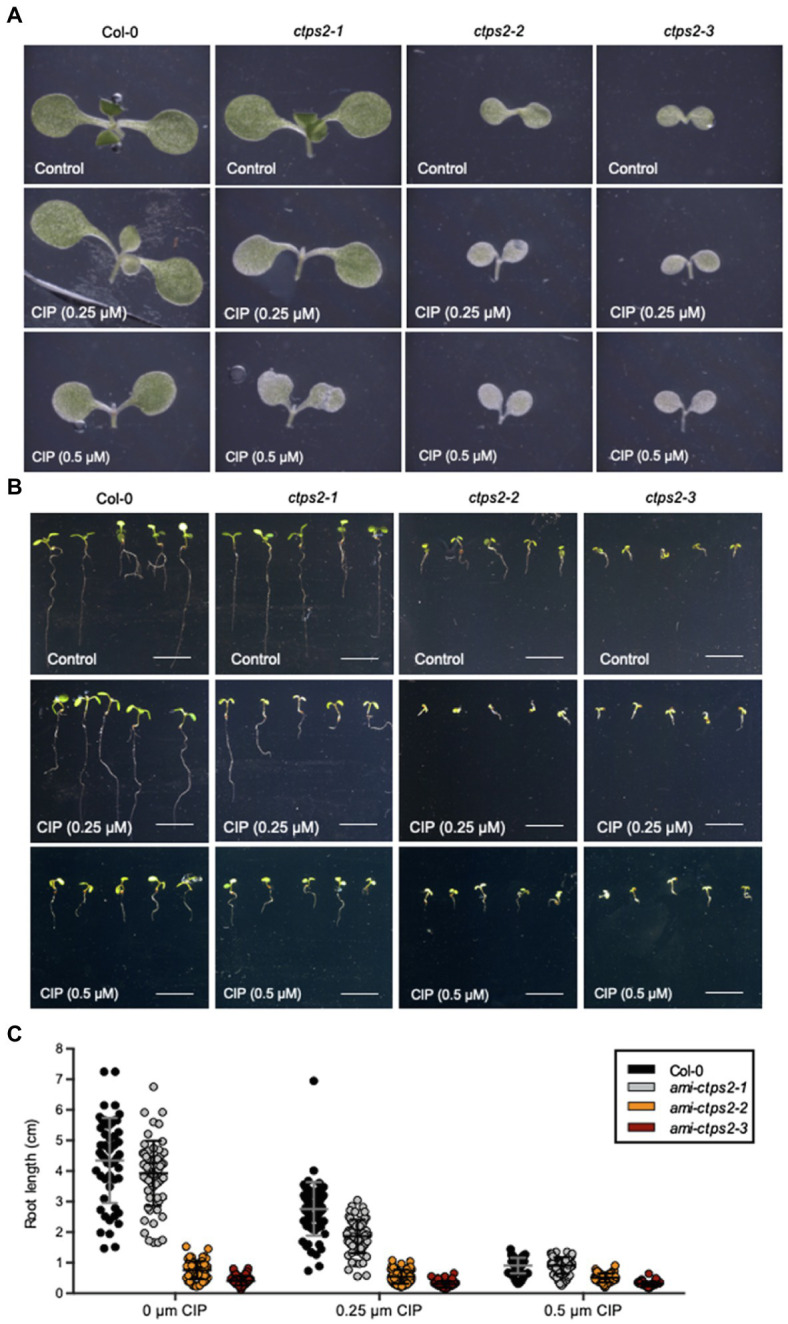
*ami-ctps2* mutants show increased sensitivity to ciprofloxacin. **(A)** Typical examples of 7-day-old *ami-CTPS2* mutant seedlings and (Col-0) control plants. **(B)** Root growth was **(C)** quantified on 7-day-old seedlings which were grown on ½ MS medium supplemented with 0, 0.25, and 0.5μM ciprofloxacin (CIP) under a 10h light/14h dark regime. Plotted are the means of at least *n*=52 biological replicates +/− SD. Scale bar in B=1cm.

Thereby, all three knock-down lines showed reduced growth after the addition of CIP (Figures 3A–C). A closer examination of the leaf tissue showed that the addition of 0.25μM CIP already led to the formation of chlorosis and to a strongly reduced growth of the *CTPS2* knock-down mutants, whereas the WT remained unchanged. Addition of 0.5μM CIP resulted in the formation of initial chlorosis in the WT. In contrast, at the same concentration of CIP, the mutants already showed complete chlorosis (Figure 3A).

To determine the effect of CIP supplementation on root growth, the root length of 7-day-old plants was measured. Already under control conditions, the CTPS2 mutants showed a strongly reduced root growth compared to the WT (Figures 3B,C). The addition of 0.25μM CIP led to a reduction of 26.4% in the WT, whereas in the *ami-ctps2-1* mutant, the root length is reduced by 52.3%. The same concentration of CIP led to a strong delay in plant development and nearly abolishes the root growths in the *ami-ctps2-2* and *ami-ctps2-3* mutants. At a concentration of 0.5μM CIP, the WT (79%) showed a similar reduction in root length as the *ami-ctps2-1* (76.9%) mutant (Figures 3B,C).

In addition, CIP treatment influenced the expression of CTPS2, as revealed by promoter studies using histochemical staining of CTPS2::GUS lines (Hickl et al., 2021). Two independent lines were inspected. All lines were germinated and grown on ½ MS media either without (control) or with the supplementation of 0.5μM CIP. Typical examples of GUS staining on 7-day-old plants are shown (Supplementary Figure 2).

Under control conditions, there was no distinct staining in either the vascular or mesophyll tissue (Supplementary Figure 2). After addition of 0.5μM CIP, there was only a slight staining of the leaf veins. Under control conditions, only root sections and the root tips were slightly colored (Supplementary Figure 2). The addition of 0.5μM CIP not only increased the intensity of the coloration but also colored larger parts of the root including tip and hair zone (Supplementary Figure 2).

**Figure 4:**
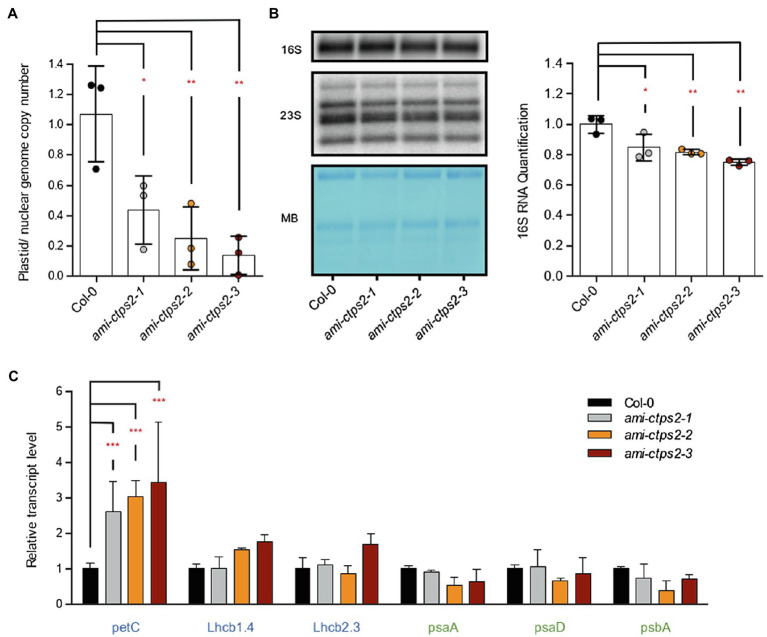
*CTPS2* is required for the maintenance of plastid genome copy number and rRNA levels. **(A)** Plastid genome copy number was determined from 28-day-old seedlings raised under a 10h light/14h dark regime. Marker genes for the plastid and nuclear genomes (three genes each) were used for comparison. Shown are the means of three biological replicates. **(B)** RNA gel-blot analysis of 16S and 23S ribosomal RNAs and quantification of 16S rRNA. The image on the left shows a representative autoradiogram and the loading control from one of three experiments (MB=Methylene blue). Band intensity was quantified with ImageJ (right panel), WT was set to 1. **(C)** Relative transcript levels of nuclear-encoded genes (*petC*, *Lhcb1.4*, and *Lhcb2.3*) and plastid-encoded genes (*psaA* and *psaD psbA*) from 28-day-old seedlings. Plotted are the means of *n*=3 biological replicates +/− SD. For statistical analysis, one-way ANOVA was performed followed by Dunnett’s multiple comparison test (*p<0.05; **p<0.01; ***p<0.001).

The observed higher sensitivity of *ami-CTPS2* lines toward CIP points to differences in chloroplast DNA amount. To test this, genomic DNA was isolated from leaves of 28-day-old plants and three plastid-encoded genes (*psaA*, *rbcL*, and *psbA*) were quantified and compared to the amount of genomic material from three nuclear-encoded genes (*Lhcb1.4*, *Lhcb2.3*, and *petC*). Compared to WT, the relative plastidic/nuclear genome copy number was significantly reduced by 56.3, 75, and 86% in *ami-ctps2-1*, *ami-ctps2-2*, and *ami-ctps2-3*, respectively (Figure 4A). In a next step, 16S ribosomal RNAs were quantified by RNA gel-blot analysis. A reduction to 84, 82, and 75% in the three ami-*ctps2* lines were observed, correlating with transcript levels of plastid-encoded photosynthesis genes (Figures 4B,C). Changes in 23S ribosomal RNA were less pronounced (Figure 4B).

To investigate the consequences of a reduced genome copy number on transcript levels of the corresponding genes, the RNA was also extracted. While the nuclear-encoded transcripts *petC* and partially also *Lhcb1.4* were increased, no significant changes in the plastid-encoded transcripts could be detected (Figure 4C). There was a tendency to reduced transcript levels in line *ctps2-2* for all three plastid-encoded genes.

### Impaired Photosynthetic Efficiency in *CTPS2* Knock-Down Mutants

**Figure 5:**
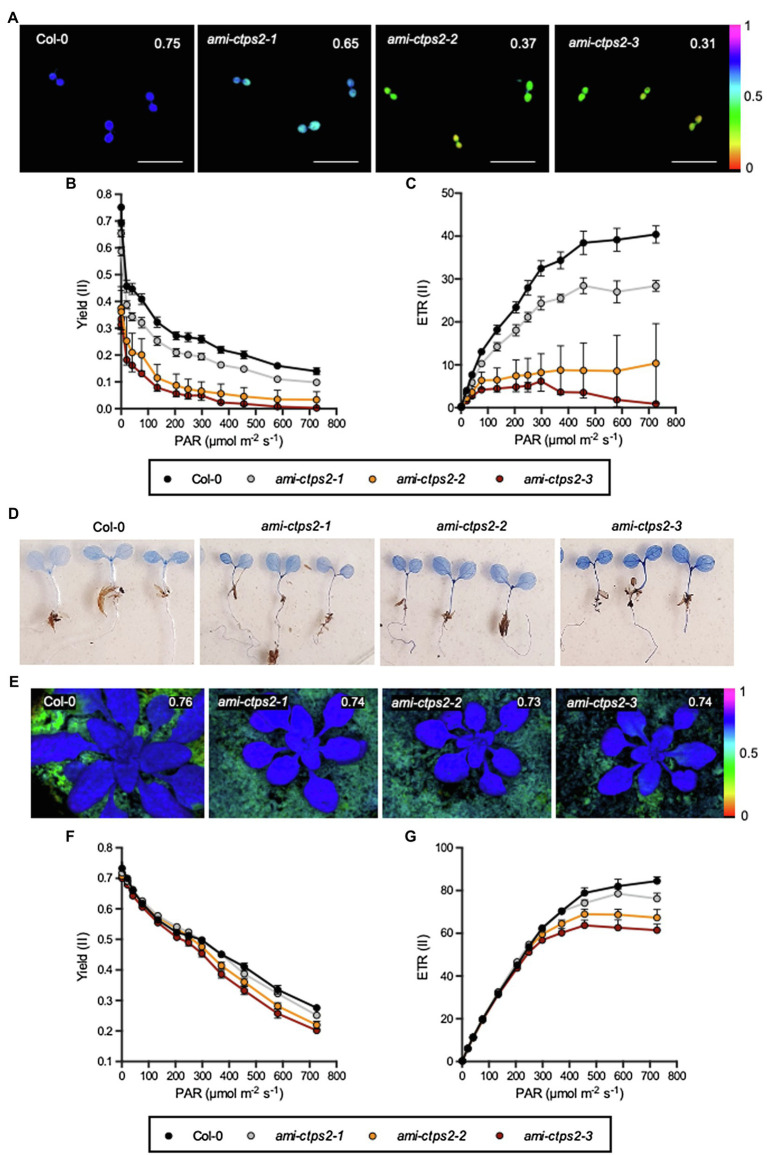
Photosynthetic performance determined by chlorophyll fluorescence measurements. **(A,E)** Graphical representation of maximum photosynthesis quantum yield, **(B,F)** effective quantum yield, and **(C,G)** electron transport rate (ETR) of 7-day-old plants **(A–C)** and 21-day-old plants **(E–G)**. **(D)** Evans-blue staining of 7-day-old seedlings. All plants were grown under a 10h light and 14h dark regime. Plotted are the means of *n*=5 biological replicates +/− SE. Scale bar in A=1cm.

The pale green phenotype of the 7-day-old *CTPS2* knock-down mutants (Figure 1C) suggests that photosynthesis is strongly affected, especially at an early stage of plant development. To investigate whether this influences the photosynthetic performance, these plants were first examined for photosynthetic parameters by using chlorophyll fluorescence imaging by pulse-amplitude-modulated (PAM) measurements in a standard light curve setting (Schreiber et al., 2007). The analysis of the maximum quantum yield (F_V_/F_M_), the effective quantum yield (Yield (II)), and the deduced electron transport rate (ETR(II)) were strongly inhibited in all three knock-down mutants (Figures 5A–C). The *ami-ctps2-1* mutants exhibited only a slight reduction (15.6%) of F_V_/F_M_, whereas the other *CTPS2* knock-down lines *ami-ctps2-2* and *ami-ctps2-3* showed a much stronger decrease of about 52 and 58.75%, respectively (Figure 5A). Similar tendencies were observed for effective yield (II) and the ETR (II; Figures 5B,C).

In order to investigate whether the energy unused by photosynthesis is released either *via* non-photochemical quenching (NPQ), or *via* nonregulated energy dissipation (NO), both parameters were determined. While the *ami-ctps2-1* line showed a slight increase in NPQ compared to the other mutant lines and the WT, we did not detect clear difference between the WT and the other two CTPS2 knock-down lines (Supplementary Figure 3A).

Opposite results were found when comparing the NO levels. The *ami-ctps2-1* line showed no significant changes compared to the WT, whereas in *ami-ctps2-2* and *ami-ctps2-3* lines, the NO levels were strongly increased (Supplementary Figure 2B). Col-0 and *ami-ctps2-1* plants reached NO values of 0.375 and 0.350, respectively, while NO was increased up to 0.490 and 0.528 in the other two CTPS knock-down lines at PAR of 134μmolm ^−2^ s^−1^, respectively.

To investigate whether increasing NPQ and NO levels lead to cell damage, 7-day-old plants were examined for membrane damage and cell death by using Evans-Blue staining. Compared with the WT control, the *ami-ctps2-1* line did not show increased staining. In contrast, the staining of *ami-ctps2-2* and *ami-ctps2-3* was more pronounced (Figure 5D).

Leaf chlorosis was not observed in 21-day-old plants. This finding went along with only slightly reduced parameters F_V_/F_M_, Yield (II), and ETR (II). Larger differences were only found when the photosystem became saturated at higher light intensities (Figures 5E–G). The analysis of the NPQ value, as well as the NO value, did not show any significant differences (Supplemental Figures 3C,D). Examinations of knock-out lines *ctps*1, 3, 4, and 5 after 7days (Supplementary Figure 3E) and 21days (Supplementary Figure 3F), respectively, indicated no changes in photosynthetic performance. This suggests that the observed defects in photosynthesis are specific for *CTPS2*.

### Vegetative Growth and Seed Development Are Impaired

**Figure 6:**
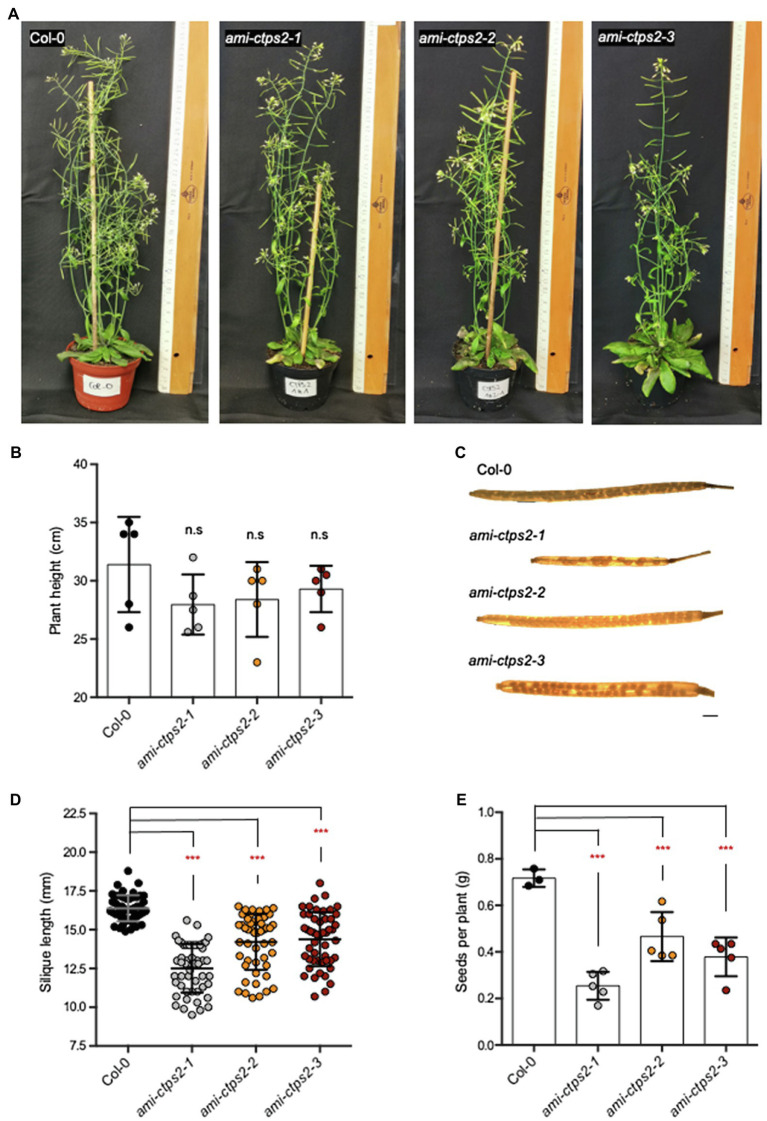
CTPS2 influences seed development. **(A)** The phenotype of 7-week-old plants grown in a 14h light/10h dark regime. **(B)** Average of the primary inflorescence height (*n*=5). **(C)** Image of siliques harvested from the plants above. **(D)** Silique length and the **(E)** amount of seeds per plant. Data points represent means ± SD. Asterisks depict significant changes between the different lines referring to Col-0 according to one-way ANOVA followed by Dunnett’s multiple comparison test (n.s=no significance; ***=*p*<0.001). Scale bar in C=1mm.

In addition to vegetative growth, *CTPS2* mutants also exhibited a severely delayed reproductive phase. Although the maximum growth height of the *CTPS2* mutants did not differ from the WT, especially, the observation of the seed pods showed clear differences. While after 7weeks most of the seeds of the WT had already matured, most of the siliques of *ami-ctps2* plants were still maturing (Figures 6A,B). When comparing the developed siliques of the WT and *ami-ctps2* plants, we found that the mutant siliques contained empty positions with missing seeds and were significantly shorter (Figures 6C,D).

As a result of these observations, significantly lower amounts of seeds per plant were obtained. While the WT produced 0.71g of seeds per plant, the *ami-ctps2-1*, *ami-ctps2-2*, and *ami-ctps2-3* mutants produced only 0.25, 0.46, and 0.38g, respectively (Figure 6E).

## Discussion

CTPS2 knock-out mutants (*ctps-2*) show an embryo lethal phenotype (Hickl et al., 2021). CTPS2 expression, however, is not restricted to embryonic tissues but is also found in leaves, roots, and flowers of Arabidopsis plants (Hickl et al., 2021). Therefore, corresponding amiRNA lines (*ami-ctps2*) were generated and the effect of CTPS2 downregulation throughout the Arabidopsis life cycle was studied. Three independent *ami-CTPS2* lines were studied, with residual *CTPS2* transcript levels ranging between 37 and 19% of WT (Figure 1). Already early in development, growth restrictions, pale leaf coloration, and impaired photosynthetic efficiency were observed in *ami-CTPS2* lines, and the severity of these parameters increased with reduced *CTPS2* expression (Figures 1, 6). In contrast, such effects were not observed for knock-out lines of the other CTPS isoforms (Figure 1; Daumann et al., 2018).

We hypothesize: downregulation of *CTPS2* (but not of any other isoform) leads to reduced chloroplast (deoxy-) nucleotide levels, further affecting chlorophyll accumulation and photosynthetic performance. This view is supported by results from a splicing mutant of *CTPS2* obtained in a suppressor screen, showing reduced chloroplast DNA amounts and impaired gene expression (Alamdari et al., 2021). When the eubacterial DNA-gyrase inhibitor ciprofloxacin, which blocks plastid DNA replication, was applied in our study, shoot and root growth of *ami-CTPS2* lines were more severely affected compared to controls (Figure 4), providing further support for this hypothesis.

To test this hypothesis, rescue experiments with cytidine and deoxycytidine were performed. Surprisingly, only the deoxy variant was effective in line with previous observations (Alamdari et al., 2021; Hickl et al., 2021). This is somewhat surprising as uptake and subsequent salvage of the nucleoside cytidine are well described in Arabidopsis (Wormit et al., 2004; Traub et al., 2007; Ohler et al., 2019). After phosphorylation and conversion to CDP, ribonucleotide reductase can then produce deoxy CDP. However, accumulation of cytidine in a cytidine deaminase mutant points to less efficient salvage. Alternatively, uptake of deoxycytidine is likely and deoxynucleotide kinase can convert deoxycytidine to dCMP (Witte and Herde, 2020). Uptake of nucleotides into chloroplasts is likely but corresponding transport activities are currently unknown (Ohler et al., 2019). However, results obtained in this work and elsewhere (Alamdari et al., 2021; Hickl et al., 2021) indicate efficient salvage and incorporation of deoxycytidine into chloroplast DNA. As dCTP levels are about 200-fold lower compared to CTP (Straube et al., 2021), adding the same amount of deoxycytidine compared to cytidine would thus more drastically affect deoxy nucleoside/nucleotide pools compared to corresponding ribo-nucleoside/−nucleotide pools. This could explain the observed higher rescue efficiency of deoxycytidine over cytidine.

The observed ciprofloxacin response altered plastid DNA copy number and rescue by deoxycytidine point to defects in DNA replication in *ami-CTPS2* mutants in line with previous findings (Alamdari et al., 2021). In Arabidopsis, CTP and GTP amounts are lowest among rNTP’s. The same holds true for dCTP and dGTP among dNTP’s (Straube et al., 2021). Therefore, increased CTP (dCTP) and GTP (dGTP) synthesis is required when the demand for new nucleic acids is high, as early in development and the establishment of photosynthesis.

But why is CTPS2 crucial for this process? In leaf tissues, CTPS2 is highest expressed among CTPS isoforms (Supplementary Table 1). CTPS4 and 5 are hardly detectable at all. CTPS3 was incapable to complement CTPS2 mutants (Alamdari et al., 2021) leaving only CTPS1 as a further candidate.

CTPS1 expression is upregulated in the two stronger *ami-CTPS2* lines, possibly as an attempt to compensate for the loss of CTPS2. Why this upregulation of CTPS1 expression and CTPS3 are unable to attenuate reduced levels of CTPS2 remains so far unclear. It could originate in different affinities of the enzymes toward substrates, activators, or feedback inhibition by CTP.

Whereas in the study by Alamdari et al. (2021) reduced transcript levels of plastid-encoded photosynthetic genes were observed, this was not the case in our work. In contrast, the expression of the nuclear-encoded petC gene, which encodes the Rieske protein of the cytochrome b6/f complex was found to be increased (Figure 3). This upregulation could be seen as an attempt to compensate reduced photosynthetic capacity of *ami-CTPS2* lines. The differences in plastid gene expression in this study compared to Alamdari et al. (2021) could originate from different growth conditions and ages of the seedlings used.

Besides reduced plastid DNA copy number, 16S rRNA content was reduced in all ami-*CTPS2* lines. As no indication for delayed RNA maturation was obtained from RNA gel-blot analysis, reduced stability or reduced synthesis could be the reason for this observation. Imbalance not only in deoxy nucleotides but also in ribonucleotides most likely contribute to a reduced plastid rRNA synthesis. Similarly, shortage of pyrimidine nucleotides in the *pumpkin* mutant leads to reduced levels of plastid rRNAs (Schmid et al., 2019).

Embryo development relies on CTPS2 activity (Hickl et al., 2021). A distinct expression pattern throughout embryo development was observed and it is likely that this contributes to the essential requirement for CTPS2. If *CTPS2* is downregulated one can assume that this will also affect embryo development in a milder form, explaining the observed differences in silique development accompanied by a reduced number of seeds per plant in all three *ami-CTPS2* lines (Figure 6).

The most marked effects were observed in *ami-ctps2-1*, although this line exhibits the highest residual *CTPS2* expression.

## Data Availability Statement

The original contributions presented in the study are included in the article/Supplementary Material, and further inquiries can be directed to the corresponding author.

## Author Contributions

LB, VS, and TM designed the research and analyzed the data. LB, VS, ED, AL, and AV performed the experiments. DH created and screened the transgenic plants. JM, LB, VS, and TM wrote the manuscript with contributions and approval from all authors. All authors contributed to the article and approved the submitted version.

## Funding

This work was funded by Deutsche Forschungsgemeinschaft (DFG grant MO 1032/5-1) to TM, DFG TR175 TP A03 to JM, and TP B08 to TM, and the Deutscher Akademischer Ausdtauschdienst (DAAD 91692277) to AV.

## Conflict of Interest

The authors declare that the research was conducted in the absence of any commercial or financial relationships that could be construed as a potential conflict of interest.

## Publisher’s Note

All claims expressed in this article are solely those of the authors and do not necessarily represent those of their affiliated organizations, or those of the publisher, the editors and the reviewers. Any product that may be evaluated in this article, or claim that may be made by its manufacturer, is not guaranteed or endorsed by the publisher.

## Footnotes

^1^ http://wmd3.weigelworld.org
