# Supplementary material for: Cytosolic CTP Production Limits the Establishment of Photosynthesis in Arabidopsis
Source: Front Plant Sci. 2021 Nov 30;12:789189. doi: 10.3389/fpls.2021.789189 (PMC8669480; doi:10.3389/fpls.2021.789189)
Supplement: Supplementary file 1 [file Data_Sheet_1.PDF]

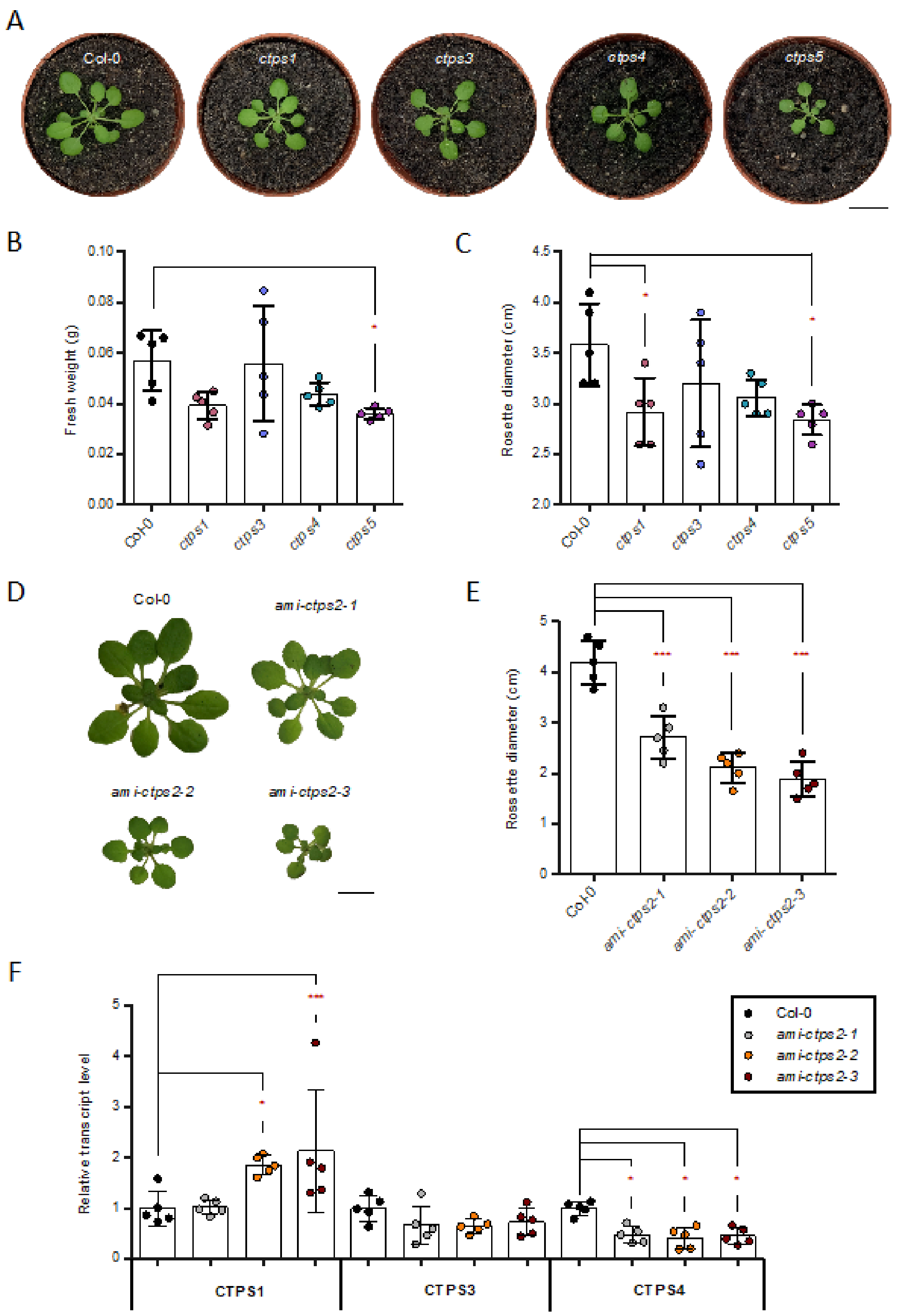

Supplemental Figure 1. CTPS2 plays a crucial role in plant development. **(A)** Shown are 28-days-old *ctps*1, 3, 4 and 5-Knock-out-plants raised under a 10h light/ 14h dark regime. (**B**) Fresh weight and (**C**) rosette diameter of the plants shown above. (**D**) Typical examples of 28-days-old CTPS2 knock-down plants with the corresponding (**E**) rosette diameter. (**F**) Relative transcript levels of CTPS1, 3 and 4 in *CTPS2* knock-down lines and the Col-0. Actin was used as a reference gene and wildtype was set to 1. Plotted are the means of n = 5 biological replicates +/- SD. For statistical analysis one-way ANOVA was performed followed by Dunnett's multiple comparison test (* p<0.05; *** p<0.001). Scale bar in **A** and **D** = 1 cm.

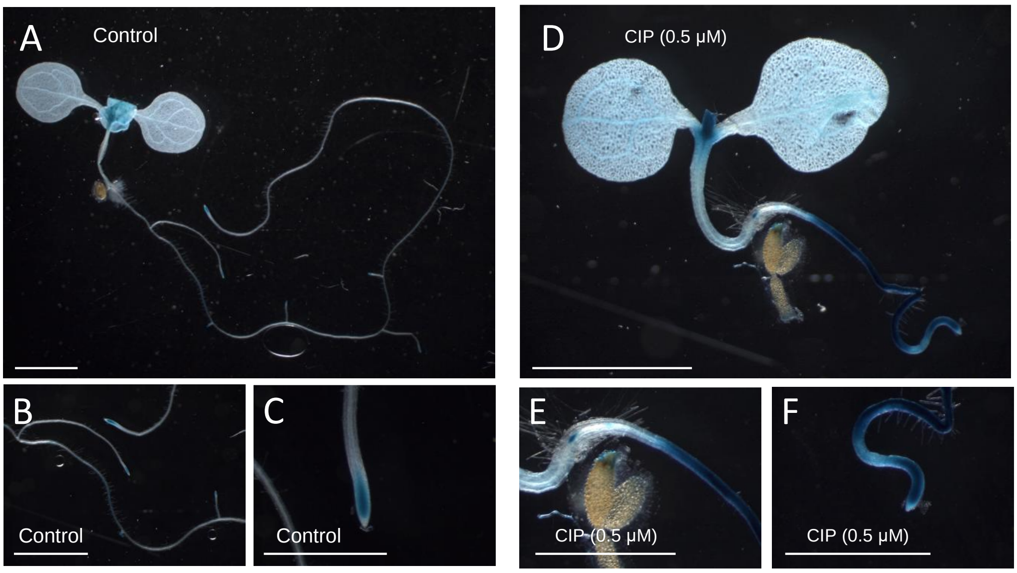

Supplemental Figure 2. Response of *CTPS2::*GUS to CIP induced genotoxic stress after histochemical staining. Seedlings were grown on ½ MS-media under a 10h light/ 14h dark regime for seven days. (**A-C**) Shows the control plants without any treatment and (**D-F**) the supplementation of 0.5 µM ciprofloxacin (CIP). Scale bar in **A**, **B** and **D** = 2 mm and in **C**, **E** and **F** = 1 mm.

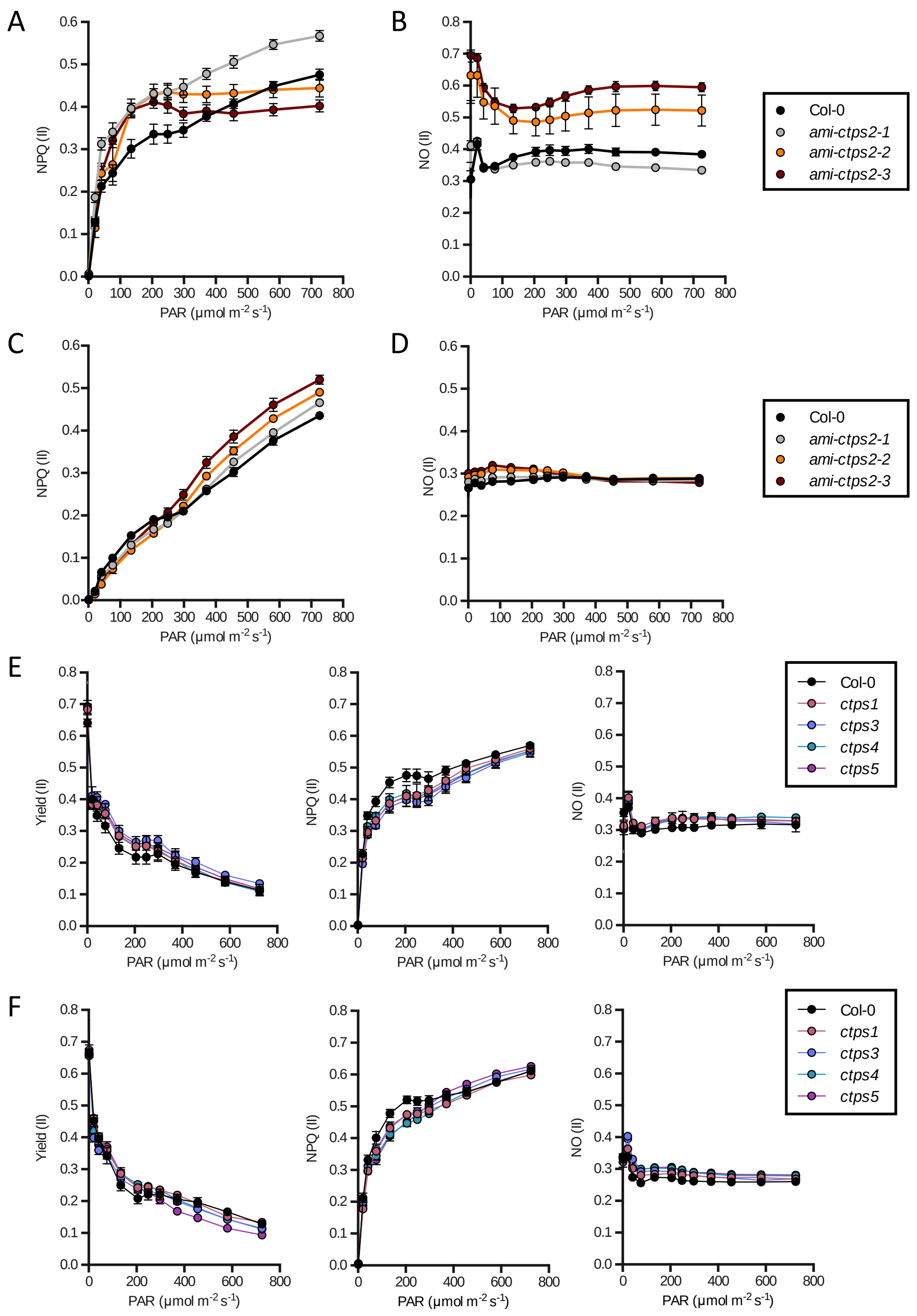

Supplemental Figure 3. Photosynthetic performance determined by chlorophyll fluorescence measurements. Quantum yield of light-induced (NPQ) and non-light-induced (NO) non-photochemical quenching of (**A, B**) seven and (**C, D**) 21-days-old CTPS2-Knockdown lines and Col-0 control plants. Effective photochemical quantum yield of photosystem II (Yield(II)), NPQ and NO of (**E**) seven and (**F**) 21-days old control plants and CTPS1-, CTPS3-, CTPS4- and CTPS5-knockout lines. All plants were grown under a 10h light and 14h dark regime. Plotted are the means of n = 5 biological replicates +/- SE.
