# Supplementary material for: Cytosolic CTP Production Limits the Establishment of Photosynthesis in Arabidopsis
Source: Front Plant Sci. 2021 Nov 30;12:789189. doi: 10.3389/fpls.2021.789189 (PMC8669480; doi:10.3389/fpls.2021.789189)
Supplement: Supplementary file 2 [file Data_Sheet_2.PDF]

Supplemental Table 1. CTPSynthase 1, 2, 3 and 4 expression levels in wildtype plants of 5 biological replicates +/- SD. Expression was normalized to expression of actin

CTPS Expression Levels	
CTPS1	0.017861 +/- 0.007718
CTPS2	0.068108 +/- 0.009135
CTPS3	0.051212 +/- 0.013179
CTPS4	0.002114 +/- 0.000299

Supplemental Table 2. List of Primers used in this work.

**Primers for cloning**	
**amiRNA-construct 1**	
I miR-s	gaTATTACCGTACAGCTTGGCTGtctctcttttgtattcc
II miR-a	gaCAGCCAAGCTGTACGGTAATAtcaaagagaatcaatga
III miR*s	gaCAACCAAGCTGTAGGGTAATTtcacaggtcgtgatatg
IV miR*a	gaAATTACCCTACAGCTTGGTTGtctacatatatattcct
**Primers for kanamycin screening**	
Ef1α_fwd	GAGACCACCAAGTACTACTGCAC
Ef1α_rev	GTTGGTCCCTTGTACCAGTCAAG
nptII_fwd	CTCGTCAAGAAGGCGATAGAAG
nptII_rev	GATGGATTGCACGCAGGTTCTC
**Housekeeping gene**	
Actin_fwd	CTTGCACCAAGCAGCATGAA
Actin_rev	CCGATCCAGACACTGTACTTCCTT
**CTPSynthase levels**	
CTPS1_fwd	GCATCGTCACAGATACGAGGTG
CTPS1_rev	CCATGCGTTTGCCAGTTTCATC
CTPS2_fwd	TGCAAGTCTGCCAAGCTGTACG
CTPS2_rev	ATCTGTGCCTGTGTCGCTCATC
CTPS3_fwd	ACAGGGAAACGTGTTGAGGTG
CTPS3_rev	GCTTCCTCGCAGCCAATATAAACC
CTPS4_fwd	TGCAAAGGAGCCTGCATTAGAGG
CTPS4_rev	TCCCAACCACAGCAATTCTTACCG
CTPS5_fwd	TAGCTTGTCGGAGCACAAAGGC

CTPS5_rev	AGAGGAATGTGCCAGATGTTGGG
**Photosynthesis related genes**	
Lhcb1.4_fwd	TCAGGCCATTGTCACCGGAAAG
Lhcb1.4_rev	TGACTGGATCAGCCAAGTGGTC
Lhcb2.3_fwd	ACTCCTCAGAGCATCTGGTACG
Lhcb2.3_rev	TTTCTGGATCGGCTGAGAGACC
psaA _fwd	AGGCTTCCACAGTTTTGGTTT
psaA_rev	CCCAAACATCTGACTGCATTT
rbcL_fwd	GCGTATGTAGCTTATCCC
rbcL_rev	TCCCCCTGTTAAGTAGTC
petC_fwd	GATGGCGATGTCAAGTGG
petC_rev	GCTTCATCTATATCCGCGTG
psbA_fwd	CTTCTGCAGCTATTGGATTGC
psbA_rev	CATTTTCTGTGGTTTCCCTGA
psaD fwd	ATGGCAACTCAAGCCG
psaD rev	CTCTTCCTGGATTCGCTTTC

RNA gel blots

16S 80-mer:

GCTATTGCCTCACCAACTAGCTAATCAGACGCGAGCCCCTCCTCGGGCGG ATTCCTCCTTTTGCTCCTCAGCCTACGGGG

23S 80-mer:

GTGGTCCTTGCTGATTCACACGGGATTCCACGTGCCCCATGCTACTCGGGTCA GAGCATAAGCTAGTGATGCTTTCGGCT

TCTACCCCTTCTTACCCTGAAAAAACAGGGACACCTTGCGTCCTTGAACCGAT AACCATCTTTCGGCTAACCTAGCCTCC

GGGGTGGGCTTACTACTTAGATGCTTTCAGCAGTTATCCGCTCCGCACTTGGC TACCCAGCGTTTACCGTGGGCACGATA
